# Improved fracture detection using the mammographic film-screen combination

**DOI:** 10.2349/biij.1.1.e3

**Published:** 2005-07-01

**Authors:** Y Faridah, BJJ Abdullah, KH Ng

**Affiliations:** Department of Biomedical Imaging (Radiology), Faculty of Medicine, University Malaya, Kuala Lumpur, Malaysia

**Keywords:** Fracture, detection, film-screen combination, mammography

## Abstract

**Aim:**

The single emulsion or single screen system is usually reserved for mammography since its use in general radiography is limited. The purpose of this study is to compare the mammographic film-screen combination (MFC) and the standard film-screen combination (SFC) in terms of fracture and soft tissue injuries detection.

**Patients, methods and materials:**

In this prospective study, 41 patients from Accident and Emergency suspected of having injury in the hands, wrists, ankles and feet regions were radiographed using both MFC and SFC. These were compared in terms of image quality, presence of fractures and soft tissue injuries. The two different film-screen combinations were also compared in terms of detection of bony fragments, film characteristics such as film speed, contrast and spatial resolution, dose and cost.

**Results:**

The MFC gives statistically better image quality compared to SFC. In 10% of patients, fractures were detected only in the MFC, which also detects tiny bone fragments that may not be resolved by the SFC. The spatial resolution of the MFC is greater than the SFC. The film speed and contrast of the MFC are lower than that of the SFC. The doses of MFC were higher compared to SFC.

**Conclusions:**

The MFC detects fractures better compared with SFC. However, the entrance skin dose for the mammographic film-screen combination was about 35% to 55% higher than the standard film-screen combination.

## INTRODUCTION

Plain radiography is an important diagnostic tool in the Accident and Emergency Department (A&E). The effectiveness of the radiograph as a diagnostic tool is firstly dependent on good radiological quality and secondly on minimization of oversight in fracture detection. On admission, 23% of fractures are overlooked on initial radiographs by radiologists resulting in mismanagement of patients. Some of these fractures were missed because of technically inadequate X-rays while others with adequate X-rays had fractures that could not be identified on admission films [1]. Clearly, a higher quality radiograph could be instrumental in achieving higher rate of detection of fractures and soft tissue injuries and hence resulting in better management.

The purpose of this study is to compare both the detection of fractures and soft tissue injuries using a mammographic film-screen combination (MFC) and the standard film-screen combination (SFC). The abilities of these two film-screen systems in detecting fractured fragments in vivo are also analyzed. The characteristics of these two film-screen systems i.e. contrast, speed, spatial resolution, entrance skin dose as well as cost were compared.

## PATIENTS AND METHODOLOGY

### Patient selection

A prospective study was carried out on patients referred from A&E, for suspected bony or soft tissue injuries involving the wrist, hand, ankle and foot. A total of 41 patients were randomly selected. Informed consent was obtained from patients or their guardian. The patients' age ranged from 17 years to 66 years with a mean of 43 years. Majority of the patients were male (80.5%). This study was approved by the hospital ethics committee.

### Image quality and detection of fractures and soft tissue injuries

A pair of radiographs using a standard radiographic combination (Kodak T-Mat G film, Kodak Lanex Regular Screen) and a mammographic film-screen combination (Fuji UM-MA HC film, Kodak Min–R Screen) was taken. For each area of interest, two identical tangential views were taken in both the mammographic and conventional film-screen combinations (Table 1).

**Table 1:** Exposure factors for standard and mammographic systems

	**Standard film-screen combination kV (mAs)**	**Mammographic film-screen combination kV (mAs)**
**Ankle**	AP 48 (5.0)	AP 70 (3.6)
	LAT 48 (4.5)	LAT 70 (3.4)
**Foot**	AP 44 (3.2)	AP 66 (3.4)
	OBL 44 (3.6)	OBL 70 (3.4)
**Wrist**	AP 44 (3.2)	AP 66 (3.4)
	LAT 44 (3.6)	LAT 70 (3.4)
**Hand**	AP 42 (2.5)	AP 63 (2.5)
	OBL 44 (2.8)	OBL 63 (2.8)

The focus-film distance (FFD) was kept at 100 cm while the film size was 24 cm × 30 cm. The radiographic factors for MFC were predetermined using a phantom while exposure factors of the SFC were those currently being used in our department (Table 1). Both types of film were then processed using the same 90s processor (Kodak M6B RPX-Omat) at a developing temperature of 32.5 deg C.

Two radiologists assessed the films independently by completing questionnaires on image quality, and detection of fractures and soft tissue injuries (Table 2). For the purpose of quality, “gold standard” images deemed to display the best image quality from each sets of SFC and MFC films were chosen as the reference [2].

**Table 2:** Questionnaire for (a) independent rating of image quality; (b) detection of fractures and soft tissue injuries

**Table:** (a) Image quality

Type of radiograph	Rating
Soft tissue	
Bone	
Cortex	
Medulla	
Joint	

Rating: 0 - non diagnostic; 1 - poor but diagnostic; 2 - textbook and diagnostic; 3 - excellent and diagnostic

**Table:** (b) Detection of fractures and soft tissue injuries

Type of radiograph	Rating
Fractures	
Soft tissue injuries	

Rating: 0 - not seen at all; 1 - seen but poor diagnostic quality; 2 - seen and of fair diagnostic quality; 3 - seen and of excellent diagnostic quality

The radiographs from SFC were assessed and rated while the radiographs from MFC were assessed two weeks later. Subsequently, another questionnaire was completed to assess the two sets of film in terms of imaging quality and detection of fractures (Table 3).

**Table 3:** Questionnaire for comparable rating of (a) image quality; (b) detection of fractures and soft tissue injuries

**Table:** (a) Image quality

Image quality	Rating
Soft tissue	
Bone	
Joint	

Rating: 1 - soft tissue seen better SFC than MFC; 2 - soft tissue seen equally well on both SFC and MFC; 3 - soft tissue seen better on MFC than SFC; 4 - bone seen better on SFC than MFC; 5 - bone seen equally well on both SFC and MFC; 6 - bone seen better on MFC than SFC; 7 - joint seen better on SFC than MFC; 8 - joint seen equally well on both SFC and MFC; 9 - joint seen better on MFC than SFC

**Table:** (b) Detection of fractures and soft tissue injuries

Detection of	Rating
Fractures	
Soft tissue injuries	

Rating: 1 - fracture seen only on SFC radiograph; 2 - fracture seen better on SFC than MFC radiograph; 3 - fracture seen equally well on both radiographs; 4 - fracture seen better on MFC than SFC radiograph; 5 - fracture seen only on MFC radiograph; 6 - no fracture seen in both radiographs; 7 - more fracture seen on MFC radiograph; 8 - more fracture seen on SFC radiograph; 9 - soft tissue injury seen only on SFC radiograph; 10 - soft tissue injury seen better on SFC than MFC radiograph; 11 - soft tissue injury seen equally well on both radiographs; 12 - soft tissue injury seen better on MFC radiograph; 13 - soft tissue injury seen only on MFC radiograph; 14 - no soft tissue injury seen in both radiograph

### In vivo detection of bone fragments

A piece of cadaveric metacarpal bone was intentionally broken into fragments of different sizes to simulate bony fractures. These bone fragments were measured in length and width (mm) and suspended in a wax mould to approximate the soft tissue density of the extremities. This phantom was exposed using both MFC and SFC. The number and size of the fragments visualized in each type of film were noted and compared.

### Assessment of film characteristics

The film characteristics i.e. the characteristic curve (H&D curve), speed, contrast, spatial resolution and dose of each film-screen combination were assessed as below.

Characteristic curve, speed and contrast – using a sensitometer, an optical density versus step number graph were obtained for both single and double film-screen combination. From this the speed, contrast and base plus fog level were obtained.

Using a resolution metal bar test object manufactured by Nuclear Associates New York, Nr 86633, the two film-screen combinations were exposed. The spatial resolution for each system was then derived.

Using a dose meter, the radiation dose in terms of mGy/mAs was calculated for the different exposures. The entrance skin dose in mGy for each region was then obtained.

## RESULTS

### Image quality and detection of fractures and soft tissue injuries

**Figure 1:**
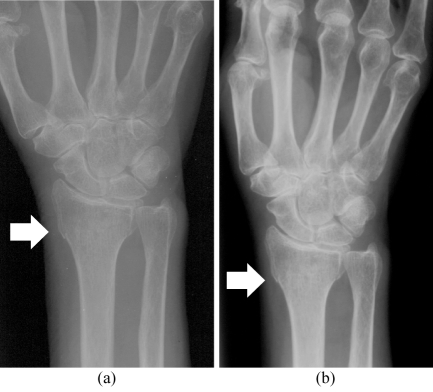
Image quality was better on the (a) MFC radiograph compared with (b) SFC radiograph. Soft tissue and outline of carpal bones are better depicted in MFC radiograph as well. Note that break in the cortex at distal radius with overlapping of fracture outline (arrow) is better appreciated in the MFC radiograph

The scores were tabulated and statistically analyzed using the Wilcoxon Signed Ranks Test [StatView software 5.0 (SAS Institute Inc. North Carolina)]. Hand and wrist radiographs were performed in 25 patients, ankle radiographs in 12 patients and foot radiograph in four patients. The hands and wrists were rated together as the size and thickness of these two regions are almost similar. The image quality of the MFC radiographs for the hands and wrists were statistically better (p<0.05) compared with the SFC radiographs (Figures 1a and 1b).

For the foot and ankle regions however, the difference in image quality of radiographs taken using the two different systems was not statistically significant.

**Figure 2:**
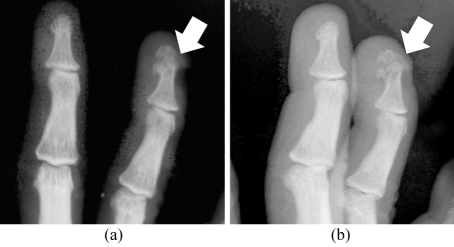
Fracture at distal phalanx of the ring finger (arrow) was better outlined due to better spatial resolution in (a) MFC compared to (b) SFC

**Figure 3:**
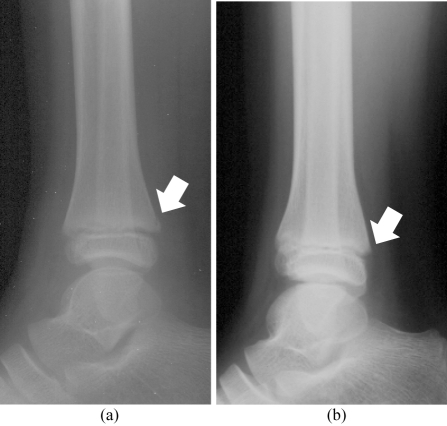
Fracture at distal posterior tibia (arrow) was only seen on MFC image (a) but was missed on SFC image (b)

In the 41 patients seen, fractures were detected in 20 patients, giving a detection rate of 48.8%. Both fractures and soft tissue injuries were statistically better detected (p<0.05) on MFC compared with SFC for the hand and wrist regions (Figures 2a and 2b). Again, the ankle and foot regions showed no statistical significance in depicting fractures and soft tissue injuries between the two film-screen combinations. Interestingly, in the 20 patients with fractures, two of these fractures were seen on the MFC but not on the SFC resulting in a 10% increase in the detection of fracture with MFC (Figures 3a and 3b).

### In-vivo detection of bone fragments

**Figure 4:**
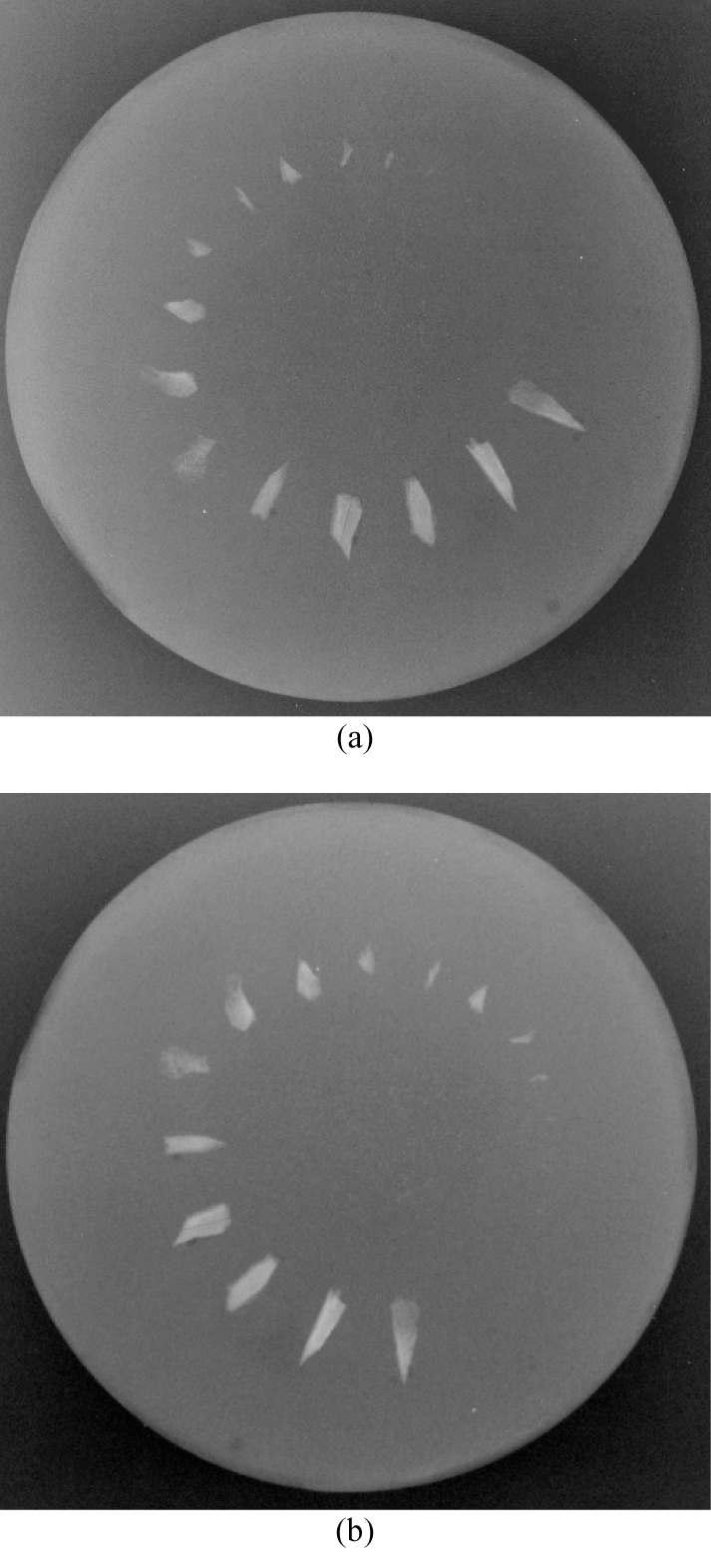
Images of bones intentionally broken then suspended in a phantom. Specks of bone fragments in the centre are better resolved in the MFC image (a) compared to the SFC image (b)

The MFC was able to resolve up to the second smallest fragment i.e. 0.7 mm x 1.0 mm whereas the SFC could only display up to the third smallest fragment measuring 1.0 mm x 2.0 mm. Furthermore the specks of bone in the centre were better appreciated in numbers and delineation on the MFC radiograph (Figures 4a and 4b).

### Film characteristics

MFC and SFC was compared in terms of film curve, film contrast, film speed, spatial resolution, entrance skin dose and cost. The base plus fog levels of these two films were almost similar i.e. 0.27 for MFC and 0.25 for SFC. The curve of the single emulsion film is steeper than the double emulsion film. The contrast index of MFC is 1.53 while the contrast index for SFC is 1.88. These are comparable to the steepness of the different curves for the different film-screen combinations seen. The contrast of MFC is comparable to SFC up to the density of 3.0. At higher densities, the contrast of SFC is higher. The speed indices for MFC and SFC are 1.20 and 2.44 respectively.

Using the resolution bar test object, the spatial resolutions of both systems were attained. The spatial resolution of MFC is 10 lp/mm whereas the resolution of SFC is 6 lp/mm.

**Figure 5:**
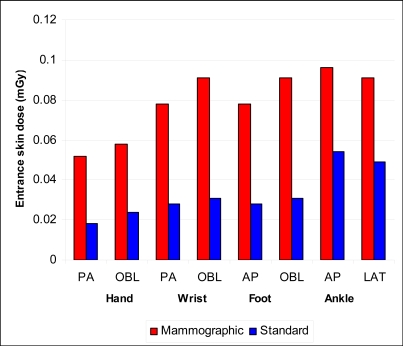
Bar chart of entrance skin doses by region of interest. There was a 34% to 40% increase in entrance surface dose in the hands and wrists region on using MFC

The entrance skin doses for MFC were higher compared to SFC in all the regions radiographed. There was a 34% to 40% increase in entrance surface dose in the hands and wrists region on using MFC. In the foot, the increase in the entrance skin dose was approximately 34%. The ankle documented the highest increase in the entrance skin dose of 54% to 57% on using MFC compared to SFC (Figure 5).

The cost of a box of 100 Fuji UM-MA HC films (single-emulsion) measuring 24 cm × 30 cm is approximately twice higher compared with the cost of a box of 100 Kodak T-Mat G films (double-emulsion) of similar dimension.

## DISCUSSION

In an outpatient setting, it had been found that the most‘missed’diagnosis is a fracture [3]. Furthermore, incorrect diagnoses were seen most frequently in the more commonly injured anatomical sites – ankles, wrist, foot, elbow and hand [4]. For example, scaphoid fractures were reported to represent 2% to 7% of all fractures and over 70% of all hand fractures presenting to accident and emergency (A&E) departments [5]. Unfortunately, conventional X-rays miss up to 2% of these fractures on the first presentation [6]. This is further compounded by the fact that emergency physicians are the first-line radiograph interpreter in an A&E setting. Rates of disagreement between emergency physicians and radiologists in the interpretation of skeletal radiographs have been documented to range from 9% to 10% [7]. A change of treatment was required for 1% to 3% of these patients [8]. Although both MRI and bone scintigraphy have been shown to display fractures earlier and with greater accuracy than conventional radiography [9], these imaging modalities are expensive and are not easily available.

Why fractures are misdiagnosed is multifactorial. Attributions have been made to poor clinical inspection or failure on the part of physician to consider certain clinical entities [10], failure to request for appropriate radiographic views [11] or failure on part of clinician to recognize a fracture on the radiograph [12]. The quality of the radiographic image also plays a part in the misdiagnosis of fractures. Review of the literature shows there are no references made to the type of the film-screen combination used in these instances where fractures were missed. Since the double film-screen combination is the most conventional system, it is fair to assume that in these cases, a SFC was employed. SFC is commercially available, has a wide exposure latitude, is easy to process and the dose to patient is reduced due to less X-ray photons used. However, SFC due to its front and back luminescent screens, give rise to lack of image sharpness. MFC, on the other hand due to its high-resolution capabilities, would make a reasonable alternative.

In comparing SFC with MFC, it was found that MFC generally gave a better image quality. This is attributed to the higher spatial resolution of MFC resulting in clearer outlines of structure. The absence of the front screen in an MFC system decreased the parallax effect, which is responsible for producing a shift in the image on two sides of film [13]. Fractures and soft tissue injuries were also better seen on MFC, where 10% of these fractures were seen only on MFC. In these two cases, the doctors attending to these patients in the A&E were informed of the fractures and proper treatment was instituted. These patients under normal circumstances would have been discharged, only to subsequently return with increased morbidity. These findings have been confirmed by the in vivo study where MFC detected small fragments of bone and specks of calcification better than SFC. A study by Oestmann et al. revealed similar results when film geometry was similar i.e. no magnification [14]. This indicated that in a clinical setting, tiny bone fragments (from an avulsion fracture perhaps) would be better detected if MFC rather than SFC was used.

The shape of the H&D curve is usually independent of the screen used and is determined only by the characteristics of the film and the processing condition [13]. Although the mammographic film is usually processed using a dedicated 2.5min processor in mammography, the film may be developed using the standard 90s processor. The base plus fog levels remained unchanged; while the speed and contrast indices were increased [15]. Film contrast is higher with SFC compared with MFC especially with densities higher than 3.0. This renders MFC with a narrow exposure latitude and thus there is not much flexibility in the exposures that can be used to form an image of optimal density. This is a major drawback, as accurate exposure factors need to be ensured before exposing the patient. This is difficult to determine as patients come in different sizes and shapes. This was not noticeable in the hand and wrist radiographs but proved detrimental in imaging of thicker parts such as the foot and ankles. Film speed is higher for SFC compared with MFC. This is because removal of the front screen in MFC decreased the number of light phosphor being emitted from the screen, but this decreased the crossover exposure effect commonly seen with SFC, significantly increasing its resolution [13, 16]. A major drawback is the increased radiation dose with MFC. This is because of the decrease in speed, which therefore increased the exposure time. There is also a concomitant increase in kVp for MFC (about 20 kV higher). The other drawback of the MFC system is its cost. Although it is slightly more expensive than SFC [15], the huge number of radiographs exposed in a large hospital everyday may incur huge costs. There would also be added costs in having to purchase new cassettes for this endeavour. In addition, the types of single film available in a country such as ours, is limited.

The paediatric patients were excluded from the study, as the exposure factors required for imaging of paediatric group would be different. It would be interesting to document whether MFC could show fractures better in this group. The study had also been limited to the extremities of upper and lower limbs. Further work with the elbow and knee regions could be carried out. This could prove beneficial especially in the knee as a number of fractures and injuries in this region are difficult to diagnose and remain undetected on SFC. The ability of MFC to display fractures and soft tissue injuries could be compared with other imaging modalities that have been shown to be superior to plain radiographs such as bone scan and magnetic resonance imaging.

## CONCLUSION

MFC gives better visualization in terms of image quality and fracture detection in the hand and wrist regions, compared with SFC. It also gives better detection of tiny bone fragments that may not be resolved by SFC. The film speed and contrast of MFC are lower than that of SFC. The spatial resolution of MFC is greater than SFC.

The radiation dose to the patient is however increased with MFC. Although radiation dose in imaging of the extremities is limited [15], patients confirmed to have fractures would need repeated radiographs and hence the radiation dose would add up considerably. Nevertheless, we believe that MFC would contribute significantly in reducing misdiagnosis of fractures as well as increasing the detection of avulsion injuries.
